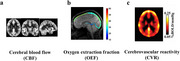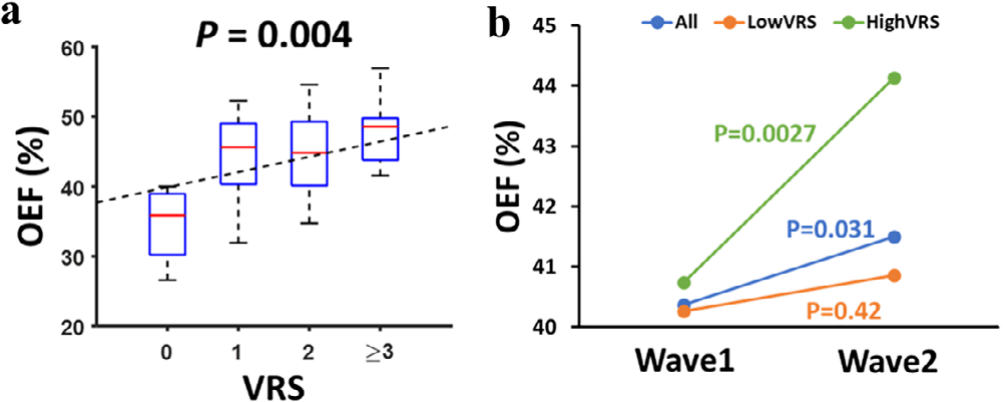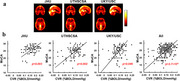# MRI‐based biomarkers of cerebrovascular function and their role in VCID

**DOI:** 10.1002/alz70856_104247

**Published:** 2025-12-24

**Authors:** Hanzhang Lu

**Affiliations:** ^1^ Johns Hopkins University School of Medicine, Baltimore, MD, USA

## Abstract

**Background:**

MRI provides an excellent tool in assessing cerebrovascular function and presents an important opportunity for the development of biomarkers in vascular contributions to cognitive impairment and dementia (VCID). In this work, we will review recent development in MRI‐based biomarkers of cerebrovascular physiology and their potential role in VCID.

**Method:**

This work will discuss 3 important parameters related to cerebrovascular function, measured with MRI (Figure 1). Section 1 will describe cerebral blood flow (CBF). CBF (Figure 1a) of the brain can be measured with a non‐contrast MRI technique referred to as arterial spin labeling (ASL). Section 2 will discuss oxygen extraction fraction (OEF). OEF (Figure 1b) reflects the balance between oxygen supply and consumption and is a known hallmark of ischemia. Section 3 will discuss a cerebrovascular reactivity (CVR) measure that reflects the brain's dynamic vascular function of vasodilatory capacity. CVR (Figure 1b) has been evaluated in the MarkVCID Consortium.

**Result:**

CBF has been investigated extensively in the context of Alzheimer's dementia. The general findings were that CBF was diminished in default model network brain regions such as posterior cingulate cortex and angular gyrus. This is thought to be related to reduced neural activity, as opposed to impaired vascular function. CBF alterations in VCID is less studied. A few studies including sporadic and genetic small vessel disease have suggested that CBF in VCID is reduced in a global fashion. OEF studies suggest that the brain's OEF is differentially affected in AD and VCID. With AD, OEF was often found to be diminished, presumably due to neurodegeneration and reduced brain metabolic rate. With VCID, OEF is elevated with higher vascular risk factors (Figure 2a) and increased faster longitudinally (Figure 2b). CVR is diminished in impaired individuals and is most strongly correlated with the Montreal Cognitive Assessment (MoCA) score (Figure 3). This association was found to be independent of AD pathological measures such as a‐beta42, tau, and ptau. CVR was also associated with executive function.

**Conclusion:**

Several MRI‐based cerebrovascular measures have shown strong promises as biomarkers in cognitive impairment and dementia, especially for VCID where available biomarkers are relatively scarce.